# Exploring the components and mechanism of *Solanum nigrum* L. for colon cancer treatment based on network pharmacology and molecular docking

**DOI:** 10.3389/fonc.2023.1111799

**Published:** 2023-03-08

**Authors:** Jin-Fang Chen, Shi-Wei Wu, Zi-Man Shi, Yan-Jie Qu, Min-Rui Ding, Bing Hu

**Affiliations:** ^1^ Institute of Traditional Chinese Medicine in Oncology, Longhua Hospital, Shanghai University of Traditional Chinese Medicine, Shanghai, China; ^2^ Department of Oncology, Longhua Hospital, Shanghai University of Traditional Chinese Medicine, Shanghai, China; ^3^ Department of Neurology, Longhua Hospital, Shanghai University of Traditional Chinese Medicine, Shanghai, China; ^4^ Department of Traditional Chinese Medicine, Ruijin Hospital, Shanghai Jiao Tong University School of Medicine, Shanghai, China

**Keywords:** colon cancer, *Solanum nigrum* L., network pharmacology, molecular docking, compounds, gene ontology, signal pathway, bioactivity

## Abstract

**Background:**

*Solanum nigrum* L. (SNL) (Longkui) is a Chinese herb that can be used to treat colon cancer. The present study explored the components and mechanisms of SNL in treating colon cancer by using network pharmacology and molecular docking.

**Methods:**

The components of SNL were collected from the TCMSP, ETCM, HERB, and NPASS databases. Meanwhile, the target proteins of these ingredients were collected/predicted by the TCMSP, SEA, SwissTargetPrediction, and the STITCH databases colon cancer-related target genes were identified from TCGA and GTEx databases. The interaction networks were established *via* Cytoscape 3.7.2. Gene Ontology and KEGG pathways were enriched by using the David 6.8 online tool. Finally, the binding of key components and targets was verified by molecular docking, and the cellular thermal shift assay (CETSA) was used to detect the efficiency of apigenin and kaempferol binding to the AURKB protein in CT26 cells.

**Results:**

A total of 37 SNL components, 796 SNL targets, 5,356 colon cancer genes, and 241 shared targets of SNL and colon cancer were identified. A total of 43 key targets were obtained through topology analysis. These key targets are involved in multiple biological processes, such as signal transduction and response to drug and protein phosphorylation. At the same time, 104 signaling pathways, such as pathways in cancer, human cytomegalovirus infection, and PI3K-Akt signaling pathway, are also involved. The binding of the four key components (i.e., quercetin, apigenin, kaempferol, and luteolin) and the key targets was verified by molecular docking. The CETSA results showed that apigenin and kaempferol were able to bind to the AURKB protein to exert anti-CRC effects.

**Conclusions:**

Quercetin, apigenin, kaempferol, and luteolin are the main components of SNL in treating colon cancer. SNL regulates multiple bioprocesses *via* signaling pathways, such as pathways in cancer, PI3K-Akt, and cell cycle signaling pathways.

## 1 Introduction

Colorectal cancer (CRC), including colon and rectal cancers, is a common malignant tumor that threatens human health within a global scope. Its incidence rate ranks top three among all malignant tumors, accounting for 10.0% of the incidence rate of all cancers, with a mortality rate of 9.4% (1). CRC can be treated with surgery, chemotherapy, radiotherapy, targeted therapy and immunotherapy. However, the effect of the treatment is always unsatisfactory. As an important treatment method for CRC, traditional Chinese medicine can inhibit cell proliferation; induce apoptosis, autophagy, and cell senescence; relieve patients’ symptoms; improve their quality of life; alleviate the toxic and side effects of chemoradiotherapy; repress metastasis and recurrence; and enhance the long-term treatment effect (2, 3).

*Solanum nigrum* L. (SNL) (Longkui) is a traditional Chinese medicine that is commonly applied to cancer treatment. SNL can inhibit the proliferation and metastatic potential of RKO CRC cells (4). SNL also induces autophagy and enhances the cytotoxicity of chemotherapy in CRC (5). With cytotoxic activity for MCF-7 cells in human breast cancer, SNL can inhibit cell migration and regulate multiple gene expressions (6). In oral cancer, SNL extracts can activate caspase-9 and caspase-3 and induce mitochondria pathway apoptosis by boosting the production of reactive oxygen species (ROS). Moreover, they can repress cell proliferation through the downregulation of cyclin-dependent kinase 1 (CDK1) and cyclin B1 (7). When applied to treat prostate cancer, SNL can arrest the cell cycle in the G2/M phase and induce cell apoptosis (8). However, the effective components of SNL and its anticancer mechanism remain to be further investigated.

Network pharmacology, an emerging discipline in recent years, can expound the relationships among “drug–gene–disease” from the aspects of the system to reveal the active components and action mechanisms of drugs (9). Chinese herbal medicinal components are complicated with diversified action characteristics and involve multiple target genes and signaling pathways. Hence, they are suitable for network pharmacology studies. In this study, the active components, targets, and related pathways of SNL in the treatment of colon cancer were explored, the “compound–target–pathway” network was established through the network pharmacology method, the key components and targets of SNL that acted on colon cancer were obtained through network topology analysis and annotated by Gene Ontology (GO) and pathway enrichment, and the binding between key components of SNL and targets was also evaluated *via* molecular docking, expecting to provide a scientific basis for the study and application of SNL.

## 2 Materials and methods

### 2.1 Identification of components of SNL

The SNL components were retrieved from the following databases: the Traditional Chinese Medicine Systems Pharmacology Database and Analysis Platform (TCMSP, http://tcmspw.com/tcmsp.php) (10), The Encyclopedia of Traditional Chinese Medicine (ETCM, http://www.tcmip.cn/ETCM/) (11), the Natural Product Activity and Species Source Database (NPASS, http://bidd.group/NPASS/index.php) (12), SymMap (http://www.symmap.org/) (13), and HERB (http://herb.ac.cn/) (14). The compound properties were inquired from the TCMSP (oral bioavailability (OB) ≥ 20%, drug-likeness (DL) ≥ 0.10, https://old.tcmsp-e.com/load_intro.php?id=29) or ETCM (drug-likeness weight (DW) ≥ 0.49).

### 2.2 Screening and prediction of compound-related targets

The target genes were retrieved from the TCMSP and ETCM. The target genes in the SNL components were also predicted using Similarity Ensemble Approach (SEA, https://sea.bkslab.org/) (15), STITCH (http://stitch.embl.de/) (16), and SwissTargetPrediction (http://swisstargetprediction.ch/) (17) based on the chemical similarity and pharmacophore model. The SMILE number of compounds was retrieved from PubChem (https://pubchem.ncbi.nlm.nih.gov/) and submitted into the SEA, STITCH, and SwissTargetPrediction databases. Next, the targets in active SNL components were predicted by taking Max Tc ≥ 0.4, confidence score ≥ 0.4, and probability value ≥ 0.5 as the criteria, and the gene name was converted into its official gene symbol.

### 2.3 Collection of colon cancer-related protein targets

The Gene Expression Profiling Interactive Analysis (GEPIA2, http://gepia2.cancer-pku.cn/#index) online tool (18) was used to identify the differentially expressed genes (fold change (FC) ≥ 2, *q*-value < 0.01) of colon adenocarcinoma (COAD) in The Cancer Genome Atlas (TCGA) and Genotype-Tissue Expression (GTEx) databases.

### 2.4 Network construction and topological analysis

The intersection set between SNL and COAD target genes was acquired using the Venn online tool (https://www.omicshare.com/tools/Home/Soft/venn), and overlapped targets were uploaded to the STRING database (https://string-db.org/) (19), the protein–protein network (PPI) was constructed, and the targets that satisfied the confidence score of ≥ 0.7 were screened out. Interaction network and topological analysis were conducted *via* Cytoscape 3.7.2 (20), including (1) the compound-target network of SNL, (2) SNL compound–target–pathway network, (3) the compound-overlapped target network, and (4) the compound–key target networks. In the compound-overlapped target network, the target genes that satisfied the degree centrality (DC) ≥ 2 × median DC, betweenness centrality (BC) ≥ median BC, and closeness centrality (CC) ≥ median CC were screened out as the key targets of SNL acted upon COAD.

### 2.5 GO and pathway enrichment analysis

The DAVID 6.8 database (https://david.ncifcrf.gov/) is an online tool for genetic function annotation (21). The key targets of SNL acted upon COAD were imported into the DAVID 6.8 database, the species were restricted to “Homo sapiens”, and the name of each target gene was converted into their official gene symbols, followed by GO (22) and Kyoto Encyclopedia of Genes and Genomes (KEGG) pathway (23–25) enrichment analysis, and visualized in online tools (http://www.bioinformatics.com.cn/, http://vip.sangerbox.com/home.html) (26).

### 2.6 Molecular docking

The protein structures were inquired about in the Protein Data Bank (PDB) (https://www.rcsb.org/) database, and the file in PDB format was downloaded (27). After the deletion of the water molecule, macromolecular ligand, and symmetric chain in PyMOL v.3.8 software, the file was saved in the PDB format (28). Operations such as hydrogenation, charge calculation, and addition of atom type were implemented for this protein file using AutoDockTools (29), and then it was saved in PDBQT format as the receptor. The SDF files of SNL compounds were retrieved from the PubChem database and optimized in the Chem3D 15.1 module of ChemOffice software, and then the SDF format was converted into mol2 format. Next, the root of the ligand was detected, and its rotatable bond was selected in AutoDockTools and exported in PDBQT format as the ligand. The affinity score was obtained by molecular docking in AutoDockTools software, and the binding site was visualized *via* the PLIP website (https://projects.biotec.tu-dresden.de/plip-web/plip/) (30) and PyMOL v.3.8 software. The active components with an affinity score ≤ −5 kJ/mol were selected as the criteria for effective binding.

### 2.7 Cytological experiment

Mouse colon cancer CT26 cells were obtained from the Cell Bank of Type Culture Collection of the Chinese Academy of Sciences (Shanghai, China) and cultured in RPMI1640 medium (Gibco, Grand Island, NY, USA) containing 10% fetal bovine serum (Gibco, Grand Island, NY, USA) in an incubator at 37°C with 5% CO_2_ and saturated humidity. Logarithmic growth stage cells were used in the experiment. Cellular thermal shift assay (CETSA) was used to detect the binding efficiency of drugs to the corresponding target proteins in CT26 cells (31, 32). CT26 cells were seeded in 10 cm culture dishes with a density of 5 × 10^5^ cells/ml. CT26 cells were collected 24 h later and washed with cold PBS three times. Cells were resuspended in RIPA lysate buffer containing protease inhibitor cocktail (Beyotime, Shanghai, China). Cell lysates were prepared by centrifugation at 12,000 rpm for 15 min at 4°C, and supernatants were collected. The supernatant was divided into three equal fractions and treated with DMSO, 100 μM Apigenin (APExBIO, Houston, USA), and 100 μM Kaempferol (APExBIO, Houston, USA), respectively. After incubating at room temperature for 1 h, the three parts were divided into nine parts (60 µl each) and heated at different temperatures (50, 55, 60, 65, 70, 75, 80, and 85°C and one aliquot kept at room temperature as control) for 3 min, followed by cooling at room temperature for 3 min. The heated lysates were centrifuged at 15,000 rpm for 20 min at 4°C, and the precipitate and soluble fraction were separated in an ice bath. The supernatant was transferred to a new centrifuge tube, analyzed by SDS-PAGE, and Western blot analysis was performed using aurora kinase B (AURKB; 1:1,000; Beyotime, Shanghai, China).

### 2.8 Statistical analysis

The data were expressed as mean ± standard deviation (SD). A two independent sample *t*-test was used when the data satisfy normality distribution and homogeneity of variance. t’-test was used when the data satisfy normality distribution but not homogeneity of variance. Non-parametric rank sum test was used for non-normally distributed data. Statistically significant differences were considered at *p* < 0.05.

## 3 Results

### 3.1 Active components of SNL

**Figure 1:**
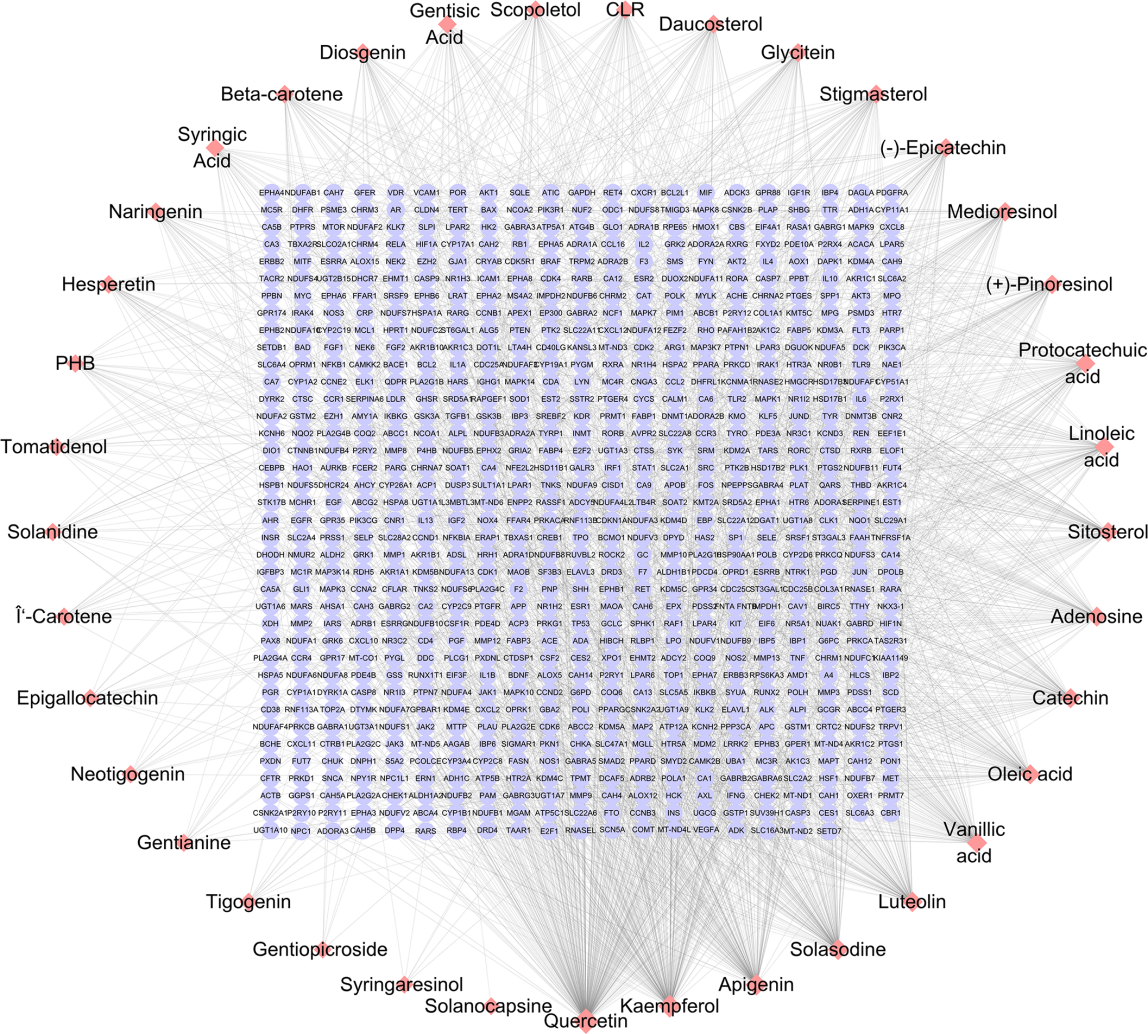
SNL component–target network. The networks were generated by Cytoscape 3.7.2 software. Pink diamond and violet round nodes represent the compounds in SNL and the potential targets of SNL, respectively. Topology analysis shows that the node size is proportional to the degree of centrality.

A total of 39 SNL components were acquired from the TCMSP database, 35 from ETCM, 114 from HERB, and 60 from NPASS. Repeated components were deleted and subjected to the OB and DL/DW screening, and then 37 effective compounds were obtained. The targets were collected and predicted in the TCMSP, STITCH, SwissTargetPrediction, and SEA databases, and 796 targets were acquired in total (**Table 1**; **Figure 1**). The components that involve over 100 target genes included quercetin, kaempferol, apigenin, and solasodine.

**Table 1:** Characteristics of SNL compounds.

Compounds	CAS	Molecular formula	Molecular weight	DL/DW	Target number
Quercetin	117-39-5	C_15_H_10_O_7_	302.25	0.28	278
Kaempferol	520-18-3	C_15_H_10_O_6_	286.24	0.24	183
Apigenin	520-36-5	C_15_H_10_O_5_	270.24	0.21	137
Solasodine	126-17-0	C_27_H_43_NO_2_	413.64	0.58	121
Luteolin	491-70-3	C_15_H_10_O_6_	286.24	0.25	98
Vanillic acid	121-34-6	C_8_H_8_O_4_	168.15	0.696	86
Oleic acid	112-80-1	C_18_H_34_O_2_	282.52	0.14	78
Catechin	154-23-4	C_15_H_14_O_6_	290.27	0.24	78
Adenosine	58-61-7	C_10_H_13_N_5_O_4_	267.24	0.495	75
Sitosterol	83-46-5	C_29_H_50_O	414.79	0.75	74
Linoleic acid	60-33-3	C_18_H_30_O_2_	278.48	0.14	72
Protocatechuic acid	99-50-3	C_7_H_6_O_4_	154.13	0.527	68
(+)-Pinoresinol	487-36-5	C_15_H_12_O_5_	358.4	0.872	61
Medioresinol	40957-99-1	C_21_H_24_O_7_	388.45	0.62	59
(-)-Epicatechin	490-46-0	C_15_H_14_O_6_	290.27	0.24	55
Stigmasterol	83-48-7	C_29_H_48_O	412.77	0.76	53
Glycitein	40957-83-3	C_16_H_12_O_5_	284.28	0.24	45
Daucosterol	474-58-8	C_35_H_60_O_6_	576.95	0.63	43
CLR	57-88-5	C_27_H_46_O	386.73	0.68	43
Scopoletol	92-61-5	C_10_H_8_O_4_	192.17	0.542	41
Gentisic Acid	490-79-9	C_7_H_6_O_4_	154.12	0.527	33
Diosgenin	512-04-9	C_27_H_42_O_3_	414.69	0.81	31
Beta-carotene	7235-40-7	C_40_H_56_	536.96	0.58	31
Syringic Acid	530-57-4	C_9_H_10_O_5_	198.17	0.762	28
Naringenin	67604-48-2	C_15_H_12_O_5_	272.25	0.742	28
Hesperetin	520-33-2	C_16_H_14_O_6_	302.28	0.27	27
PHB	99-96-7	C_7_H_6_O_3_	138.12	0.539	25
Tomatidenol	546-40-7	C_27_H_43_NO_2_	413.64	0.58	19
Solanidine	80-78-4	C_27_H_43_NO	397.64	0.563	18
Î’-Carotene	7488-99-5	C_40_H_56_	536.96	0.58	16
Epigallocatechin	970-74-1	C_15_H_14_O_7_	306.27	0.27	14
Neotigogenin	470-01-9	C_27_H_44_O_3_	416.64	0.81	12
Gentianine	439-89-4	C_10_H_9_NO_2_	175.18	0.601	12
Tigogenin	77-60-1	C_27_H_44_O_3_	416.64	0.611	11
Gentiopicroside	20831-76-9	C_16_H_20_O_9_	356.32	0.39	6
Syringaresinol	21453-69-0	C_22_H_26_O_8_	418.4	0.737	6
Solanocapsine	639-86-1	C_27_H_46_N_2_O_2_	430.7	0.67	1

### 3.2 GO and pathway enrichment analysis of SNL targets

**Figure 2:**
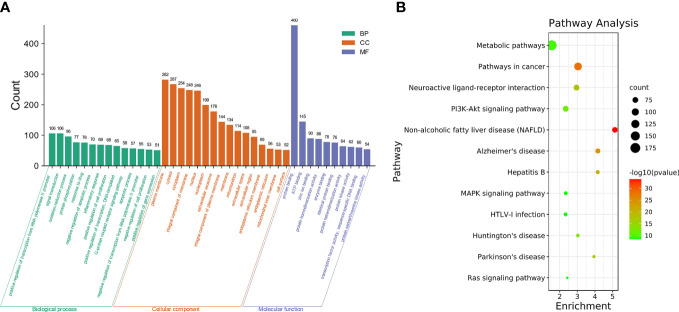
GO and pathway enrichment of SNL targets. **(A)** GO enrichment of SNL targets (count number ≥ 50). **(B)** KEGG pathway enrichment analysis of SNL targets (count number ≥ 50).

**Figure 3:**
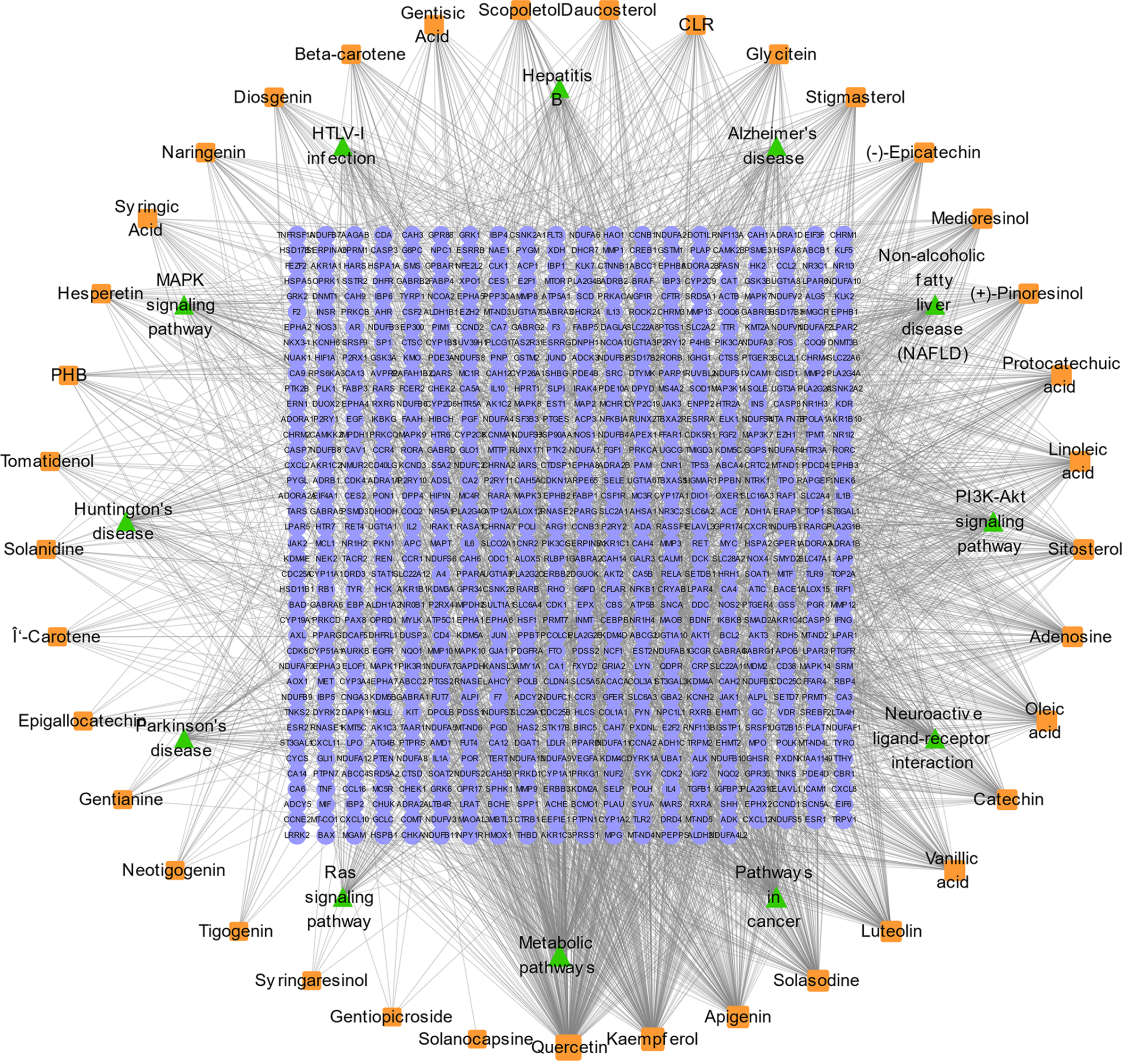
SNL compound–target–pathway network. SNL compound–target–pathway (count number ≥ 50) networks were generated by Cytoscape 3.7.2 software. Light blue ellipse, orange round, and green triangle nodes stand for SNL targets, SNL compounds, and pathways, respectively.

The GO and KEGG enrichment analyses (**Figures 2A, B**) were performed using the DAVID 6.8 online tool. The results showed that the targets of SNL mainly existed in cell regions, such as the plasma membrane, cytoplasm, nucleus, membrane, mitochondrion, endoplasmic reticulum, and mitochondrial inner membrane, with their molecular functions involving the binding to protein, ATP, zinc ion, enzyme, and sequence-specific DNA. Meanwhile, they were correlated with the activity of transcription factors and protein kinases and took a part in bioprocesses, such as signal transduction, oxidation–reduction process, protein phosphorylation, drug response, inflammatory response, gene expression, cell proliferation, and apoptotic processes (**Figure 2A**). According to the KEGG pathway analysis results, a total of 148 pathways were affected by the active components of SNL (*p <* 0.05), and those ranking in the top 12 (gene number ≥ 50) included metabolic pathways, pathways in cancer, neuroactive ligand–receptor interaction, PI3K-Akt signaling pathway, non-alcoholic fatty liver disease (NAFLD), Alzheimer’s disease, hepatitis B, MAPK signaling pathway, HTLV-I infection, Huntington’s disease, Parkinson’s disease, and Ras signaling pathway (**Figures 2B**, **3**).

### 3.3 Identification of SNL targets against COAD

**Figure 4:**
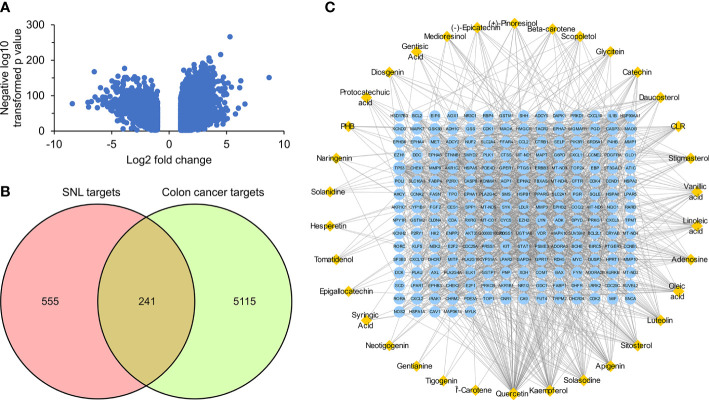
Overlapped targets of SNL and colon cancer. **(A)** Differently expressed genes in colon cancer were identified by the GEPIA2 online tool from TCGA and GTEx databases and expressed as volcano plots. **(B)** Shared targets of SNL and colon cancer. **(C)** SNL compound-shared target networks. Topology analysis shows that the node size is proportional to the degree of centrality.

The GEPIA2 online tool screen showed 5,356 genes (i.e., 2,682 upregulated genes and 2,674 downregulated genes) differentially expressed in COAD (**Figure 4A**; **Table 2**). The Venn analysis showed 241 overlapped targets of SNL and COAD (**Figure 4B**). Through the PPI analysis in the STRING database, 215 targets showed high interactions (confidence score ≥ 0.7). A compound-overlapped target network, which consisted of 215 nodes and 930 edges, and the node size was in direct proportion to the degree of centrality, was constructed *via* Cytoscape 3.7.2. By the topological analysis of this network, 43 key targets and 254 interactions were acquired, and all the targets presented high interactions (confidence score ≥ 0.7) (**Figure 4C**; **Table 3**). The key targets related to the main SNL components are listed in **Table 4**, where the components with a number of key targets of ≥ 5 were quercetin, apigenin, kaempferol, luteolin, adenosine, solasodine, sitosterol, and linoleic acid.

**Table 2:** Identified the top 10 up- and downregulated genes in colon cancer.

Upregulated genes			Downregulated genes		
Gene name	log_2_ FC	Adjusted *p*-value	Gene name	log_2_ FC	Adjusted *p*-value
RP11-40C6.2	8.703	2.92E−197	DES	−8.427	9.31E−102
CEACAM6	6.596	8.47E−107	MYH11	−7.068	2.41E−104
DPEP1	6.243	2.18E−181	ACTG2	−7.012	1.41E−90
S100P	6.145	5.74E−163	SYNM	−6.771	9.04E−110
LCN2	6.027	1.99E−91	ADH1B	−6.516	3.75E−214
CEACAM5	5.776	1.52E−70	RP11-394O4.5	−6.452	2.41E−128
CLDN2	5.429	3.97E−166	CNN1	−6.243	5.10E−88
ETV4	5.297	0.00E+00	HSPB6	−6.176	1.16E−116
CDH3	5.258	0.00E+00	ADAM33	−5.97	2.11E−160
MMP7	5.129	1.45E−181	LMOD1	−5.782	7.09E−127

**Table 3:** Key targets of SNL that act on colon cancer.

Targets	Official name	Degree	BC	CC
TP53	Cellular tumor antigen p53	68	0.419	0.510
HSP90AA1	Heat shock protein HSP 90-alpha	33	0.062	0.405
MYC	Myc proto-oncogene protein	32	0.043	0.410
CXCL8	Interleukin-8	29	0.043	0.385
CCND1	G1/S-specific cyclin-D1	28	0.024	0.405
PIK3R1	Phosphatidylinositol 3-kinase regulatory subunit alpha	27	0.048	0.376
CASP3	Caspase-3	26	0.047	0.422
CDK1	Cyclin-dependent kinase 1	24	0.007	0.377
CXCL12	Stromal cell-derived factor 1	23	0.042	0.396
LPAR1	Lysophosphatidic acid receptor 1	23	0.021	0.352
CCNA2	Cyclin-A2	23	0.006	0.371
MMP9	Matrix metalloproteinase-9	23	0.099	0.416
LPAR2	Lysophosphatidic acid receptor 2	22	0.011	0.339
CCNB1	G2/mitotic-specific cyclin-B1	21	0.006	0.374
GAPDH	Glyceraldehyde-3-phosphate dehydrogenase	21	0.068	0.420
ADCY2	Adenylate cyclase type 2	20	0.014	0.319
CXCL10	C-X-C motif chemokine 10	20	0.008	0.340
LPAR5	Lysophosphatidic acid receptor 5	20	0.004	0.331
GPR17	Uracil nucleotide/cysteinyl leukotriene receptor	20	0.004	0.331
LYN	Tyrosine-protein kinase Lyn	20	0.033	0.361
FYN	Tyrosine-protein kinase Fyn	20	0.016	0.352
CDK2	Cyclin-dependent kinase 2	20	0.006	0.375
CTNNB1	Catenin beta-1	20	0.018	0.393
CHRM2	Muscarinic acetylcholine receptor M2	19	0.014	0.333
ADCY5	Adenylate cyclase type 5	19	0.010	0.318
CXCL2	C-X-C motif chemokine 2	18	0.003	0.327
IL1B	Interleukin-1 beta	18	0.057	0.370
FGF2	Fibroblast growth factor 2	18	0.014	0.408
ADORA3	Transmembrane domain-containing protein TMIGD3	17	0.009	0.325
PLK1	Serine/threonine-protein kinase PLK1	17	0.004	0.368
CDK4	Cyclin-dependent kinase 4	17	0.014	0.377
EPHA2	Ephrin type-A receptor 2	16	0.038	0.379
STAT1	Signal transducer and activator of transcription 1-alpha/beta	16	0.012	0.391
HSPA8	Heat shock cognate 71 kDa protein	16	0.032	0.385
GSK3B	Glycogen synthase kinase-3 beta	15	0.009	0.380
PSME3	Proteasome activator complex subunit 3	15	0.009	0.366
E2F1	Transcription factor E2F1	15	0.004	0.366
BCL2L1	Bcl-2-like protein 1	14	0.005	0.385
AURKB	Aurora kinase B	14	0.011	0.351
CCL2	C–C motif chemokine 2	14	0.011	0.392
EPHB2	Ephrin type-B receptor 2	14	0.009	0.342
PPARG	Peroxisome proliferator-activated receptor gamma	13	0.039	0.386
EZH2	Histone-lysine *N*-methyltransferase EZH2	13	0.021	0.365

**Table 4:** SNL compounds and related key targets.

Compound	Number of key targets	Targets
Quercetin	22	AURKB, BCL2L1, CASP3, CCL2, CCNB1, CCND1, CDK1, CDK2, CXCL10, CXCL2, CXCL8, E2F1, GSK3B, HSP90AA1, IL1B, MMP9, MYC, PIK3R1, PLK1, PPARG, STAT1, TP53
Apigenin	9	TP53, BCL2L1, CASP3, CCNB1, CCND1, CDK1, GSK3B, MMP9, PSME3
Kaempferol	8	CASP3, CDK1, CHRM2, GSK3B, HSP90AA1, MMP9, PPARG, STAT1
Luteolin	7	ADCY2, CASP3, CCNA2, CCNB1, CDK2, GSK3B, MMP9
Adenosine	6	ADCY5, ADORA3, EZH2, GAPDH, GPR17, HSPA8
Solasodine	5	AURKB, EZH2, FGF2, GSK3B, LYN
Sitosterol	5	CASP3, CHRM2, EPHA2, EPHB2, FGF2
Linoleic acid	5	CHRM2, LPAR1, LPAR2, LPAR5, PPARG
Glycitein	4	CCNA2, CDK4, GSK3B, PPARG
Oleic acid	4	LPAR1, LPAR2, LPAR5, PPARG
Beta-carotene	3	CASP3, CTNNB1, MYC
Vanillic acid	2	CXCL12, FYN
(-)-Epicatechin	2	CASP3, CCL2
Stigmasterol	2	CHRM2, EPHA2
Daucosterol	2	EPHA2, EPHB2
CLR	2	EPHA2, EPHB2
Catechin	1	HSP90AA1
Protocatechuic acid	1	FYN
Scopoletol	1	CASP3
Diosgenin	1	TP53
Naringenin	1	CCL2
Epigallocatechin	1	CXCL8

### 3.4 GO enrichment analysis of SNL key targets

**Figure 5:**
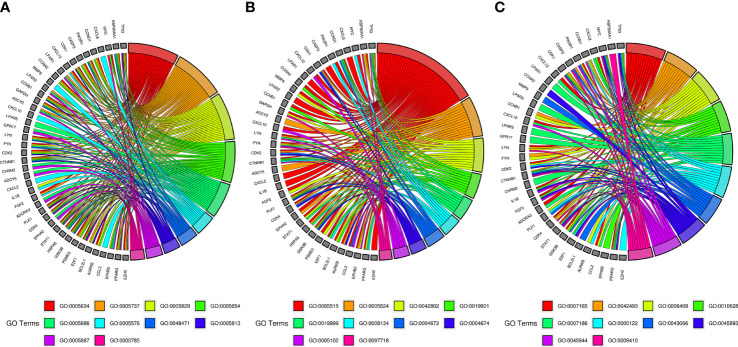
GO enrichment analysis of SNL key targets. **(A)** Cellular component enrichment. GO:0005634, nucleus; GO:0005737, cytoplasm; GO:0005829, cytosol; GO:0005654, nucleoplasm; GO:0005886, plasma membrane; GO:0005576, extracellular region; GO:0048471, perinuclear region of cytoplasm; GO:0005813, centrosome; GO:0005887, integral component of plasma membrane; GO:0000785, chromatin. **(B)** Molecular function enrichment. GO:0005515, protein binding; GO:0005524, ATP binding; GO:0042802, identical protein binding; GO:0019901, protein kinase binding; GO:0019899, enzyme binding; GO:0008134, transcription factor binding; GO:0004672, protein kinase activity; GO:0004674, protein serine/threonine kinase activity; GO:0005102, receptor binding; GO:0097718, disordered domain-specific binding. **(C)** Biological process enrichment. GO:0007165, signal transduction; GO:0042493, response to drug; GO:0006468, protein phosphorylation; GO:0010628, positive regulation of gene expression; GO:0007186, G-protein-coupled receptor signaling pathway; GO:0000122, negative regulation of transcription from RNA polymerase II promoter; GO:0043066, negative regulation of apoptotic process; GO:0045893, positive regulation of transcription, DNA templated; GO:0045944, positive regulation of transcription from RNA polymerase II promoter; GO:0009410, response to a xenobiotic stimulus.

GO and pathway enrichment analyses of key targets were carried out *via* DAVID6.8 online tool. The GO analysis manifested that these targets mainly existed in regions, such as the nucleus, cytoplasm, cytosol, nucleoplasm, plasma membrane, extracellular region, perinuclear region of cytoplasm, centrosome, an integral component of the plasma membrane, and chromatin (**Figure 5A**). They could also bind to molecules, such as protein, ATP, protein kinase, enzyme, transcription factor, and receptor, which were related to the activity of protein kinase and tyrosine/serine/threonine kinase (**Figure 5B**). Furthermore, they participated in various bioprocesses, such as signal transduction, response to drug, protein phosphorylation, positive regulation of gene expression, G-protein coupled receptor signaling pathway, regulation of transcription from RNA polymerase II promoter, negative regulation of the apoptotic process, positive regulation of transcription, DNA-templated, and response to xenobiotic stimulus (**Figure 5C**).

### 3.5 Pathway enrichment analysis of SNL key targets

**Figure 6:**
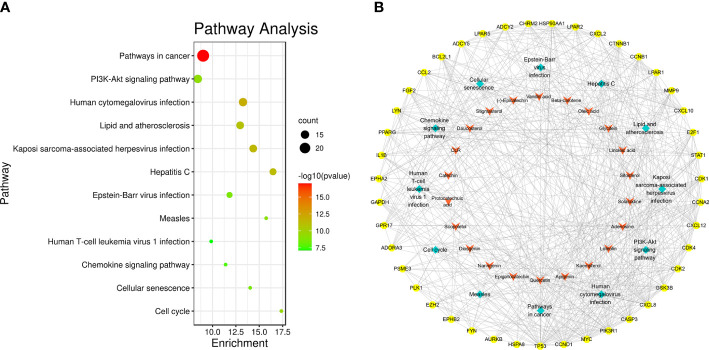
Pathway enrichment analysis of SNL key targets. **(A)** KEGG pathway enrichment. **(B)** Network of the compound–key target–pathways. Yellow octagon, red V, and cyan diamond nodes stand for key targets, compounds, and pathways, respectively.

**Figure 7:**
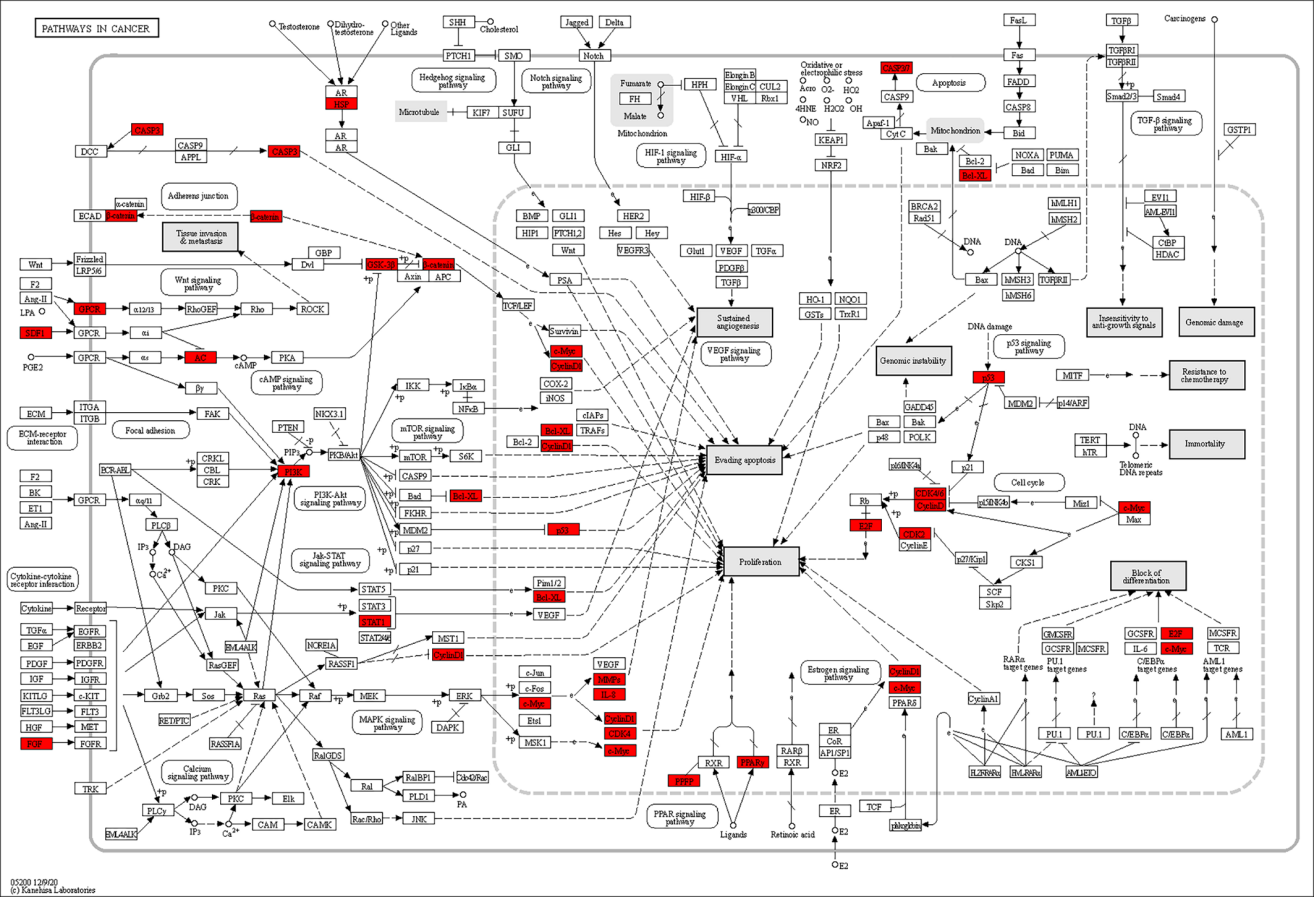
Distribution of key targets in pathways in cancer. The red rectangles stand for the key targets. (KEGG Copyright Permission 230270).

According to the pathway enrichment analysis, 104 pathways, including pathways in cancer, human cytomegalovirus infection, PI3K-Akt signaling pathway, Kaposi sarcoma-associated herpesvirus infection, lipid and atherosclerosis, hepatitis C, Epstein–Barr virus infection, cell cycle, measles, cellular senescence, chemokine signaling pathway, and human T-cell leukemia virus 1 infection, were affected by key targets (*p <* 0.05) (**Figures 6A, B**). The key targets distributed in pathways in cancer are displayed in **Figure 7**.

### 3.6 Molecular docking

**Figure 8:**
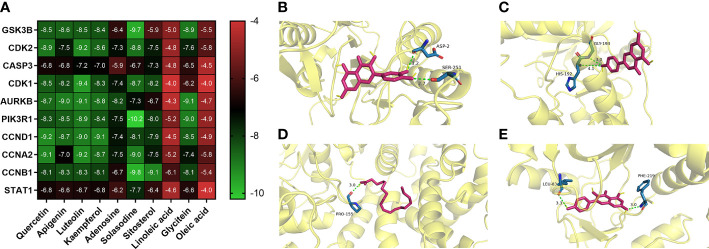
Representative molecular docking. **(A)** Binding affinity score of key compounds and key targets. **(B)** Quercetin binding with CASP3. **(C)** Apigenin binding with AURKB. **(D)** Linoleic acid binding with CCNA2. **(E)** Kaempferol binding with AURKB.

Quercetin, apigenin, kaempferol, and luteolin were selected and docked with the corresponding key targets by using the AutoDockTools software. The binding sites between compounds and key targets were visualized using the PLIP website and PyMOL v.3.8 software. The lower the binding energy between ligand and receptor, the stabler their binding conformation would be. By screening according to the criterion of affinity score ≤ −5 kJ/mol, four active components (i.e., quercetin, apigenin, kaempferol, and luteolin) could stably bind to the corresponding key targets. Quercetin and CASP3 formed two hydrogen bonds through ASP-2 and SER-251. Apigenin and AURKB formed two hydrogen bonds through HIS-192 and GLY-193. Linoleic acid and CCNA2 formed a hydrogen bond through PRO-155. Kaempferol and AURKB formed two hydrogen bonds through LEU-83 and PHE-219. All of them form two hydrogen bonds each, except for linoleic acid and CCNA2, which form one hydrogen bond. The findings above indicated that quercetin, apigenin, and kaempferol are probably the core active components of SNL (**Figure 8**).

### 3.7 CETSA

**Figure 9:**
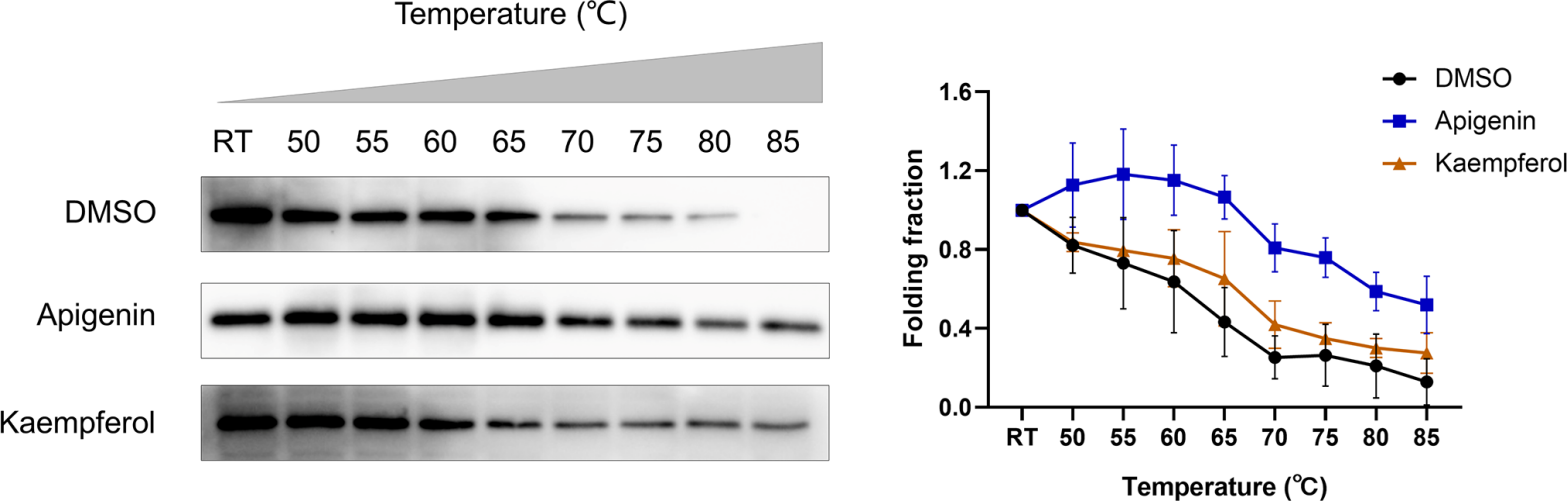
Cellular thermal shift assay (CETSA). CETSA was performed in CT26 cells treated with apigenin (100 μM), kaempferol (100 μM), or DMSO control. The effect of apigenin and kaempferol on AURKB protein stability was evaluated by Western blot assay. RT, room temperature.

The stability of compound-induced target proteins was examined by CETSA, and the interaction of apigenin and kaempferol with AURKB in CT26 cells was determined by a western blot assay. As shown in **Figure 9**, the expression of the AURKB protein decreased continuously with the increase in temperature. The heat stability of the AURKB protein in CT26 cells was significantly higher than that in the control group under the intervention of 100 μM apigenin and 100 μM kaempferol. Taken together, these biophysical binding characteristics suggested that AURKB may be a direct target of apigenin and kaempferol.

## 4 Discussion

SNL is a commonly clinically used anticancer in traditional Chinese medicine. The antitumor effect of SNL is mainly manifested by repressing the proliferation, arresting the cell cycle, inducing cell apoptosis, and inhibiting the cell migration of tumor cells (33–36). However, the active components of SNL and their mechanisms remain unclear. The components and potential targets of SNL were explored through the network pharmacology method. The study results showed that SNL targets existed in multiple cellular areas, involved diversified molecular functions, and were correlated with pathways, such as metabolic pathways, pathways in cancer, neuroactive ligand–receptor interaction, PI3K-Akt signaling pathway, non-alcoholic fatty liver disease (NAFLD), Alzheimer’s disease, hepatitis B, MAPK signaling pathway, HTLV-I infection, Huntington’s disease, Parkinson’s disease, and Ras signaling pathway, suggesting that SNL exerts extensive pharmacological effects in cancers, nervous system diseases, non-alcoholic fatty liver disease, HBV, and HTLV-I virus-related diseases.

The results showed that compounds, such as quercetin, apigenin, kaempferol, luteolin, adenosine, solasodine, sitosterol, and linoleic acid, were the active components of SNL, and quercetin, apigenin, kaempferol, and luteolin were the key components of SNL in treating colon cancer. Quercetin, apigenin, kaempferol, and luteolin are all natural flavonoids that can be used as anticancer agents (37–40). Darband et al. found that quercetin has potential preventive properties in CRC by inhibiting proliferation, metastasis, and angiogenesis, inducing apoptosis, and regulating cell metabolic activities and related signaling pathways (41). In HCT116 cells, apigenin downregulated the expression of Cyclin B1 and its activating partners, Cdc2 and Cdc25c, induced poly (ADP-ribose) polymerase (PARP) cleavage, decreased the expression of procaspase-3, procaspase-8, and procaspase-9, and upregulated the level of LC3-II, suggesting that apigenin could regulate the cell cycle and induce apoptosis in HCT116 cells by inhibiting autophagy (42). Kaempferol has a significant antiproliferative and cytotoxic effect on HCT116, HCT15, and SW480 cells, which could promote the cleavage of PARP in HCT116 and HCT15 cells as well as the activation of caspase-8, caspase-9, and caspase-3, phosphor-p38 MAPK, p53, and p21. These changes were reversed after treatment with the ROS inhibitor NAC, the pan-caspase inhibitor z-vad-fmk, and the p38 MAPK inhibitor SB203580. The above results suggested that p38 phosphorylation and caspase activation mediated by ROS and p53 signaling are closely related to kaempferol-induced apoptosis in CRC cells (43). Luteolin has no effect on the proliferation of CRC cells but could inhibit the migration and invasion of CRC cells by downregulating MMP2, MMP9, and MMP16 *in vitro* and *in vivo*. The expression of miR-384 was downregulated and that of pleiotrophin (PTN) was upregulated compared with adjacent normal tissues, suggesting that the antitumor effects of luteolin were partly mediated through the miR-384/PTN axis (44)..

In addition to the four abovementioned key components, other components also had anti-CRC effects. Linoleic acid facilitates CRC cell apoptosis by inducing oxidative stress and mitochondrial dysfunction (45). Sitosterol activated caspase-9 and caspase-3 and boosted the CRC cell apoptosis by inhibiting PI3K/Akt and regulating the expressions of Bcl-xl and Bad (46). Solasodine induced the CRC cells’ apoptosis by regulating AKT/GSK3B/β-catenin signaling pathway and activating the caspase cascade (47). By regulating the IL-6/STAT3 pathway, beta-carotene repressed the polarization of M2 macrophages and inhibited the fibroblasts activated by transforming growth factor-β1 (TGF-β1) to inhibit the invasion and migration and the epithelial–mesenchymal transition of CRC cells, as well as azoxymethane/dextran sodium sulfate-induced CRC formation (48).

In this study, 43 key targets of SNL were found for the treatment of colon cancer. As serine/threonine kinase, AURKB was able to regulate the G2/M phase of the cell cycle in cell mitosis, and its overexpression was related to the CRC prognosis (49). Glycogen synthase kinase 3 beta (GSK3B) is a multifunctional serine/threonine kinase involved in mediating various cellular functions such as proliferation, apoptosis, metabolism, differentiation, and cell motility (50). Rosales-Reynoso et al. discovered that GSK3B may play a role in tumor progression by regulating the Wnt/β-catenin pathway and that GSK3B gene variation is associated with tumor location and tumor-node-metastasis (TNM) stage in CRC patients (51). Caspase-3 (CASP3) is an apoptotic executive protein, which can not only inhibit the invasion and metastasis of CRC cells but also increase the sensitivity of tumor cells to radiotherapy and chemotherapy (52). During cell division, cyclin-dependent kinase 2 (CDK2) is a core cell cycle regulator that is active from the late G1-phase to the S-phase. Overexpression of CDK2 leads to abnormal regulation of the cell cycle, which is directly related to the overproliferation of tumor cells. Thus, CDK2 inhibitors could induce antitumor activities (53, 54). CCND1, a type of cyclin, was correlated with the cell cycle and proliferation. Studies have shown that miR-374a could inhibit the PI3K-Akt pathway by lowering the expression of CCND1 to inhibit the proliferation of CRC cells (55). Transcription 1 (STAT1) can repress miR-181a, thereby further inhibiting the phosphatase and tensin homolog (PTEN)/Akt signal transduction and the proliferation of CRC cells (56). In addition, the molecular docking results showed that the key SNL components, namely, quercetin, apigenin, luteolin, and kaempferol, presented favorable binding activity to the corresponding key targets, such as GSK3B, CDK2, and CASP3. Subsequent CETSA experiments validated these results, showing that AURKB could bind to apigenin and kaempferol and perform the corresponding biological functions.

According to the results of GO and pathway enrichment analysis, the key targets of SNL can bind to protein, ATP, protein kinase, enzyme, transcription factor, and receptor; regulate the activities of tyrosine/serine/threonine kinases; influence pathways in cancer, human cytomegalovirus infection, PI3K-Akt signaling pathway, Kaposi sarcoma-associated herpesvirus infection, lipid and atherosclerosis, hepatitis C, Epstein–Barr virus infection, cell cycle, measles, cellular senescence, chemokine signaling pathway, and human T-cell leukemia virus 1 infection and further took a part in bioprocesses, such as signal transduction, response to the drug, protein phosphorylation, regulation of the apoptotic process, gene expression, and transcription, DNA-templated, and response to a xenobiotic stimulus.

The pathways in cancer and colorectal cancer were the sets of multiple pathways, including MAPK, p53, PI3K-Akt, Wnt, and Jak-STAT signaling pathways. Mitogen-activated protein kinases (MAPKs) are serine-threonine kinases that could link extracellular signals to fundamental cellular processes such as cell growth, proliferation, differentiation, migration, and apoptosis (57). The MAPK pathway was closely related to the adhesion, angiogenesis, invasion, and migration of CRC cells (58). The Janus kinase (JAK)-signal transducer of activators of transcription (STAT) pathway participated in the proliferation, invasion, and migration of tumor cells, whereas miR-198 could inhibit the proliferation and induce the apoptosis of CRC cells by repressing the JAK-STAT signal transduction (59). The phosphatidylinositol-3 kinase (PI3K)/protein kinase B (Akt) signaling pathway, an important tumor cell pathway, participated in gene transcription and translation, and it was related to the cycle, proliferation, apoptosis, and autophagy of CRC cells (60). As a cancer suppressor gene, p53 could regulate the downstream genes and take a part in DNA repair and regulation of the cell cycle and apoptosis (61). Reactivation and restoration of p53 function hold great potential for the treatment of CRC. The Wnt signaling pathway participates in the regulation of cell cycle, proliferation, and apoptosis and has been suggested as a therapeutic target for CRC treatment (62).

## 5 Conclusion

In conclusion, the present study demonstrated that the active components of SNL for the treatment of colon cancer include quercetin, apigenin, kaempferol, and luteolin, and the effective targets include AURKB, PIK3R1, CDK1, CDK2, GSK3B, CCNA2, CCND1, CCNB1, CASP3, CCNA2, and STAT1. SNL regulates bioprocesses such as signal transduction, response to the drug, protein phosphorylation, gene expression, and apoptotic process *via* signaling pathways, including pathways in cancer, the PI3K-Akt signaling pathway, cell cycle, cellular senescence, and the chemokine signaling pathway. The CETSA confirmed that apigenin and kaempferol, the active components of SNL in colon cancer treatment, were able to bind to AURKB. The present study provides a basis for the treatment of CRC by SNL from the perspective of network pharmacology. We identified the main components, targets, and signaling pathways of SNL for the treatment of colon cancer and provided new clues for the R&D of anti-CRC drugs.

## Data availability statement

The datasets used and/or analyzed during the current study are available from the corresponding author on reasonable request.

## Author contributions

Conception and design: JFC and BH. Administrative support: BH. Provision of study materials: JFC and BH. Collection and assembly of data: JFC, SWW, YJQ and MRD. Data analysis and interpretation: JFC. All authors contributed to the article and approved the submitted version.
